# Study of the Frequency and Specificity of Red Cell Antibodies in Patients with Hemoglobinopathies

**DOI:** 10.1007/s12288-023-01651-4

**Published:** 2023-04-17

**Authors:** Manal M. Wilson, Manal M. W. El Masry, Mona Kamal El-Ghamrawy, Nessma Abd El-Hadi, Amany A. Abou-Elalla

**Affiliations:** 1 Departments of Clinical and Chemical Pathology, Faculty of Medicine, Cairo University, Cairo, Egypt; 2 Pediatric Haematology, Faculty of Medicine, Cairo University, Cairo, Egypt; 3 Technology of Medical Laboratory Department, Faculty of Applied Health Science, Misr University for Science and Technology, Cairo, Egypt

**Keywords:** Multitransfused, Alloimmunization, Antibodies, Blood transfusion, sickle cell disease, Thalassaemia

## Abstract

Patients with thalassemia and sickle cell disease (SCD) require blood transfusions as part of their supportive care. However, one of the most serious side effects of this treatment is the risk of red cell alloimmunization. The goal of this study was to assess the prevalence and Specificity of red cell alloimmunization in Egyptian thalassemia and sickle cell anaemia patients. This study included 200 multi transfused Egyptian patients, one hundred and forty patients with transfusion dependent thalassaemia and sixty patients with sickle cell anaemia, who were attending the Paediatric Children Hospital-Cairo University at the period from March 2019 to October 2019. Alloantibody identification was made by Diamed- ID microtyping system. In the studied groups both thalassemia and sickle patients, the prevalence of alloimmunization was 22/200 (11%) patients. The two most often alloantibodies were, antibodies against Kell antigen (37%) and against E antigen (30%). The prevalence of alloimmunization was more in females in comparison to males, but it did not reach statistical significance and patients with thalassemia major had higher alloimmunization rates than other studied groups but was not statistically significant. In the D negative patients in the research group, alloimmunization demonstrated a statistically significant difference (*p* = 0.01). Age, gender, age of transfusion onset and splenectomy were not contributing factors to the antibody presence in the group of patients being investigated. Before receiving blood transfusions, extended red blood cell phenotyping should be thought of as a crucial procedure for hemoglobinopathies patients who would likely have several transfusions. It is advised that haemoglobinopathies patients in Egypt be checked through phenotyping of RBC units for Kell and all Rh antigens to be phenotyped before starting transfusion in these patients which is also standard of care for these patients presently.

## Introduction

Haemoglobinopathies are the most prevalent, clinically significant single gene disorders worldwide. In Egypt β-thalassemia is the most frequent haemoglobinopathy, with a carrier rate of 6–10%. Each year an estimated 1000 infants born with β-thalassaemia major, among the national 1.5 million live births [1].

For severe cases of thalassemia, appropriate and regular red cell transfusions remain the mainstay of treatment; nevertheless, transfusion-related complications such as viral infections, hemosiderosis and RBC antigen immunization restrict its benefits [2].

Alloantibodies and autoantibodies development, is one of the transfusion related problems that can complicate transfusion therapy, studies reported some alloantibodies are haemolytic causing haemolytic transfusion reactions, while others are clinically insignificant [3].

In addition to reducing RBC post-transfusion survival, the development of alloantibodies may make it more difficult to obtain suitable cross-match compatible blood [2]. These antibodies can increase the necessity for blood transfusion, in patients with haemoglobinopathies.

The immunomodulatory effect of isogenic blood transfusion on the recipient’s defence system, erythrocyte antigenic variation between donor and recipient, and the recipient’s immune state are all effective factors for alloimmunization. It prevents safe blood transfusions, causes infantile haemolytic illnesses, and produces a variety of haemolytic transfusion reactions [4].

The reported worldwide alloimmunization frequency rate among thalassemia patients varies from 1.13 to 40.4%. The most common alloantibodies reported were antibodies against Rh (C, c, & E), Kell (K), Kidd (Jka & Jkb), and Duffy (Fya & Fyb) RBC antigens [5].

It is unclear what causes alloimmunization in thalassaemia patients. But some research revealed that the recipient’s immune status, lack of a spleen, and different red cell phenotype between donors and recipients are likely to contribute even more to the phenomenon [6].

### Aim of Work

Beta-thalassemia and SCD are both present in Egypt, with the former being more common, however there is no national screening programme, and even carrier detection by premarital and/or early antenatal screening for both thalassemia and SCD is not mandatory and not widely applied in Egypt. β-Thalassemia and SCD create both a social and financial burden for the patients’ family and the Egyptian government. The high frequency of β-thalassemia carriers with increasing rate of newly born cases, is a demanding reason for studying any factor that can affect thalassemia health. So we decided to study the frequency, types and risk factors that contribute to development of alloantibodies in multitransfused haemoglobinopathies. And give these patients their types of alloantibodies they have, aiming for future safe blood transfusion.

## Patients and Methods

### Patients

This study was conducted in accordance with the code of conduct of research in Egypt and with the 1964 Helsinki Declaration and its later amendments. It was approved by the Research Ethics Committee of the Department of Clinical and Chemical pathology, Cairo University. Informed written consent was obtained from the patients or their legal guardians for all participants. Every patient was informed that all information gathered for this study would be kept completely private and would not be utilised for any other purpose.

A cross sectional study was conducted in the Paediatric Children Hospital-Cairo University at the period from March 2019 to October 2019, for a total of 200 regularly transfused haemoglobinopathies, previously diagnosed. Patients in need of blood transfusion for other haemolytic or systemic disease were excluded from the study. Our patients were 107 males and 93 females. Their ages ranged between 1 and 39 years with a mean age of 12 years.

### Transfusion Protocol

All patients were transfused with non leukodepleted, ABO and Rh (D) compatible packed red blood cells, with the aim to keep target Hb level 9–11.5 g/dl.

Clinical data, including age, sex, age of start of transfusion, transfusion frequency and splenectomy were collected from all patients.

### Methods

All patients were subjected to complete blood count and blood grouping (ABO, RH typing/reversed grouping system) using (DiaMed Cassetes & centrifuge). Each patient was tested for detection of autoantibodies and alloantibodies using screening and identification techniques.

Antibody screening test was carried out using 3 RBCs panel (Diamed), The results were interpreted according to the manufacturer’s instructions. Cases showing positivity in antibody screening were subjected to antibody identification using 11 RBC panel (Diamed), which is based on Column Agglutination Technology. An auto control was also done simultaneously to determine the presence of autoantibody.

### Statistical Methods

Data were statistically described in terms of frequencies (number of cases) and percentages and compared using Chi square (χ2) test. Exact test was used instead when the expected frequency is less than 5. All statistical calculations were performed using computer program SPSS (Statistical Package for the Social Science; SPSS Inc., Chicago, IL, USA) release 15 for Microsoft Windows (2006).

## Results

### Patient Characteristics

Our study included 200 haemoglobinopathies patients, 107 (54%) males and 93 (46%) females with an age of 1–39 years (mean age 12y). Thalassemia major comprised 100 (50%) cases, thalassemia intermedia 40 (20%) of cases and 60 (30%) cases had sickle cell anaemia. The patient’s demographic data, transfusion history and clinical data are summarized in (Table 1).

**Table 1:** Demographic and clinical data of all studied patients*

Variable	Thalassemia (*n* = 140) (major 100/ intermedia 40)	Sickle cell anaemia (*n* = 60)	Total (*n* = 200)
*Gender*			
Male (*n*/%)	74 (53)	33 (55)	107 (54)
Female (*n*/%)	66 (47)	27 (45)	93 (46)
*Age*			
< 12 (*n*/%)	69 (49)	35 (58)	104 (52)
≥ 12 (*n*/%)	71 (51)	25 (42)	96 (48)
*Age of transfusion onset*			
< 1 year (*n*/%)	125 (89)	10 (17)	135 (68)
≥ 1 year (*n*/%)	15 (11)	50 (83)	65 (32.5)
* Frequency of transfusion/year *			
< 12/year (*n*/%)	31 (22)	43 (72)	74 (37)
≥ 12/year (*n*/%)	109 (78)	17 (28)	126 (63)
*Spleen status*			
Splenectomized (*n*/%)	75 (54)	20 (33)	95 (48)
None splenectomized (*n*/%)	65 (46)	40 (67)	105 (52)

*Data are reported as number (%)

The predominant blood group, was A positive, 72 (36%), followed by 62 (31%) had O positive, 42 (21%) had B positive, 19 (9.5%) had AB positive, 3 (1.5%) had O negative, one (0.5%) had A negative and one (0.5%) had B negative. RhD was negative in 5 (2.5%) of our cases.

### Antibody Screening and Identification of Alloantibodies

Alloantibodies were detected in 22 (11%) patients, most of them had only one alloantibody 19 (86.4%), while one patient (4.5%) had two antibodies and two patients (9.1%) had three alloantibodies.

These alloantibodies were detected in 13/100 (13%) of the thalassemia major cases and in 3/40 (7.5%) of the thalassemia intermedia cases. Regarding sickle cell cases, alloantibodies were detected in 6/60 (10%) of the studied group.

A total of 27 alloantibodies with 9 different subtypes were identified in both studied groups (thalassemia and sickle cell patients) in our cohort. The most frequent antibody was anti-K in 10/27 (37%), followed by anti E 8/27 (30%), anti-C 2/27 (7%), anti D 2/27(7%), anti c, 1/27(4%), anti-Kidd 1/27 (4%), anti-Cw 1/27 (4%), anti-Duffy 1/27 (4%) and anti M in 1/27 (4%).

In patients with Thalassemia the majority of red cell alloantibodies developed were against anti- K 8/17(47%), followed by anti-E 4/17 (23.5%), anti-D 2/17 (11.8%), anti-c 1/17 (5.9%), anti-Cw 1/17 (5.9%), and anti M 1/17 (5.9%), (Table 2).

**Table 2:** The number and specificity of alloantibodies in alloimmunized thalassemia patients*

Type of antibody	Frequency	
	Number	Percent (%)
* RH*		
Anti D	2	11.8
Anti E	4	23.5
Anti C^w^	1	5.9
Anti c	1	5.9
* Kell*		
Anti K	8	47
* MNS*		
Anti M	1	5.9

*Data are reported as number (%)

While in sickle cell anaemia cases the majority of red cell alloantibodies were against. anti-E 4/10 (40%), followed by anti-K 2/10 (20%), anti-C 2/10 (20%), anti-Kidd 1/10 (10%), and Anti-Duffy 1/10 (10%), (Table 3).

**Table 3:** The number and specificity of alloantibodies in alloimmunized sickle cell anaemia patients*

Type of antibody	Frequency	
	Number	Percent (%)
* RH*		
Anti C	2	20
Anti E	4	40
* Kell*		
Anti K	2	20
* Duffy *		
Anti Fya	1	10
* Kidd*		
Anti Jka	1	10

*Data are reported as number (%)

**Table 4:** Frequency of alloantibodies in the studies patient’s in relation to demographic and clinical data

Parameter	Alloantibodies*		*P* value
	Present *n* (%)	Absent *n* (%)	
* Gender *			
Male Female	10 (9) 12 (12)	97 (91) 81 (87)	0.432
* Age *			
< 12 years ≥ 12 years	9 (9) 13 (13)	95 (91) 83 (86)	0.366
* Diagnosis *			
Thalassemia major Thalassemia intermedia Sickle cell	13 (13) 3 (8) 6 (10)	87 (87) 37 (92) 54 (90)	0.616
* Age of transfusion onset *			
< 1 year ≥ 1 year	15 (11) 7 (11)	120 (89) 58 (89)	0.942
* Frequency of transfusion /year*			
< 12 units ≥ 12 units	9 (12) 13 (10)	65 (88) 113 (90)	0.687
* Splenectomy*			
Yes No	14 (15) 8 (8)	81 (85) 97 (92)	0.108
*Laboratory data*			
ABO system A B O AB	11 (15) 3 (7) 7 (11) 1 (5)	62 (85) 40 (93) 58 (89) 18 (95)	0.46
*RH phenotyping*			
Positive Negative	19 (10) 3 (60)	176 (90) 2 (40)	**0.01**

*Data are reported as number (%)

Bold value indicates statistically significant (*P* < 0.05)

### Comparison of the Demographic and Clinical Data of All the Studied Patients in Relation to the Frequency of Alloantibodies

Table 4 shows the differences between alloimmunized and non alloimmunized cases regarding the risk factors which are prone to cause alloantibodies formation, briefly, there was no statistically significant difference between the two groups, regarding patient’s gender, age, diagnosis, age of transfusion onset, frequency of transfusion, splenic condition and ABO blood group. However, there was strong statistical significant difference (*P* = 0.01) regarding the Rh status of the studied group. Autoantibody was detected in only three (1.5%) patient.

## Discussion

Quality of life and life expectancy for haemoglobinopathies patients are dramatically improved by regular blood transfusion and iron chelation [7]. Alloimmunization is one of the serious hazards of transfusion (SHOTs) [8], that can complicate RBC cross matching, shortens RBC survival, delays efficiency of safe transfusion and accelerates tissue iron loading [9].

Alloimmunization rates are variable in haemoglobinopathies patients. In the current study, the frequency of alloimmunization was 11%. This was similar to the study of Ahmed et al., (2010) who reported an incidence of 11.3% in Egyptian patients with Thalassemia and sickle cell anaemias [10]. However higher frequency was recorded in other Egyptian studies as El-Beshlawy et al. [2] who reported an incidence of 18% in Thalassemia patients. This difference could be attributed to the inclusion of Sickle cell anaemia patients in our study whose age of onset and frequency of transfusions could be lower than in Thalassemic patients [9]. Geographical variations also could be a contributing factor in alloimmunization frequency, as higher frequencies of 23.1, 23.8 and 26% in Mansoura, Menoufia and Tanta respectively. Their studies were performed in the Northern Goverenates of Egypt which historically have been subjected to occupation by other civilizations, as Roman and Greek empires. This could have an impact on the genetic make-up of the residents in these Northern areas [11–13]. This observation could be supported by the lower incidence of alloimmunization in multitransfused thalassemic patients in the Meditteranean basin. In Greece, Politis et al. [14], reported an incidence of 11.6% of alloimmunization in his study on Thalassemia and sickle patients despite pretransfusion RBC phenotyping that is routinely performed.

Racial diversity could also be a contributing factor as Singer et al. [3] reported an incidence of 20.8% among Asian patients in the United States despite proper pre-transfusion screening procedures. In Taiwan, Wang et al. [15] reported an incidence of 37% of alloimmunization in thalassemia patients with transfusion therapy. Similarly, Pazgal et al. [16] in his study in Israel, reported an incidence of 42.5% of alloimmunization in forty transfusion dependent Thalassemia patients that were included in his study. Despite routine RBC antigen screening is performed in these areas, however, the high rate of alloimmunization could be attributed to low prevalence RBC antigens which may have a crucial role in multi-transfused patients. In support of this hypothesis is a study made by El Danasoury et al. [17] who compared the incidence of alloimmunization between thalassemia transfusion dependent patients included in a limited donor exposure program (LDEP) and patients receiving donation from multiple donors. There was a significant drop in alloimmunization rates in LDEP group (8.3%) compared to non LEDP one (21.6%).

On comparing, the incidence of alloimmunization in patients with thalassemia (11.4%) and sickle cell anaemia (10%) in our study, there was no statistical significant difference in alloimmunization frequency between the two groups. However, Alkindi et al. [18] reported that patients with sickle cell anaemia (31.6%) showed a higher rate of alloimmunization than in thalassaemia patients (20%) with a statistically significant difference. This difference could be attributed to the larger number of sickle patients (133) included in his study, compared to our sickle cell anaemia group (60 patients). Another contributing factor, could be the early onset of blood transfusion in thalassemia patients frequently compared to sickle cell anaemia. Murao et al. [19] reported a similar finding which he attributed to the younger age of onset and frequency of blood transfusions in thalassemia patients compared to sickle cell patients. Early onset of transfusions could lead to immunological tolerance to red cell alloantigen in younger patients.

In the current study, alloimmunization was detected in 22 patients (11%) of the 200 patients included in the study. Thalassemia patients showed alloimmunization in 16/140 (11.4%) patients while in sickle cell anemia 6/60 (10%) patients developed alloantibodies. Regarding the type of alloantibodies detected in our work, a total of 27 alloantibodies with 9 different subtypes were identified in both studied groups (thalassemia and sickle cell patients) in our cohort. The most frequent alloantibody in patients with thalassemia was anti K 8/17 (47%) followed by anti E 4/17 (24%). Similar to another study in Egypt, Osman et al. [12] reported the highest frequency of alloantibodies was anti K (37.3%) and anti E antigens (34.3%) in thalassemia patients. In other countries the same finding was reported as in Israel, Oman, Palastine and Greece [16, 18, 20, 21]. However, in contrast to our results, Abdelrazik et al. [22] study in Egypt recorded that anti-D was the most frequent alloantibody found in (4.25%), followed by anti-C, anti c and anti- E having a similar incidence of 1.1% respectively. Anti Kell antibodies was detected in only one patient despite being the most common alloantibody in several studies. The authors attributed this difference as they conducted their study in Fayoum governorate which is characterized by a low population diversity and most of the donors and recipients are from the same ethnic group. Our study was performed in Cairo which hosts many diverse communities with a variable donor pool.

Regarding the alloantibodies detected in patients with sickle cell anaemia, the most frequent alloantibody was anti E found in 40%, followed by anti K in 20% and anti C 20% of the studied group. Al Dawood et al. [23] reported antibodies to Rh E, K, Rh C, and Rh c were the most encountered, with a prevalence of 34.6, 30.8, 14, and 11.2% of alloimmunized SCD patients, respectively. Both our results matched most of the studies in the literature, [18, 24, 25]. Evers et al. [26] emphasized their immunogenicity and capability to stimulate the humoral immune response. This provides further evidence that strict prophylactic partial RBCs matching for Rh and K antigens for all SCD patients might reduce the incidence of alloimmunization [27].

In our studied group (thalassemia and sickle cell anaemia patients), five patients had Rh negative phenotype. Interestingly, three of those patients had anti D alloantibody denoting alloimmunization to the D antigen. This could be caused by the accidental transfusion of donor blood with a weak Rh variant which was mistyped as Rh negative during routine typing. The epitopic diversity of Rh D variants can mount a sufficiently strong immune response in recipients with different variants and thus being responsible for the formation of anti-D alloantibodies [17]. This emphasisis the need of use advanced molecular techniques for the proper typing of Rh negative donors to ensure safe blood transfusion. Similar to this result, alloimmunization to D antigen was significantly higher in RH negative patients in other studies in USA, Egypt and India [9, 22, 28].

Another independent risk factor associated with alloimmunization is the patient’s age with significantly higher alloimmunization risk in the older age group. This is due to the development of acquired immune tolerance to red cell alloantigen’s in young children [29]. This finding was previously stressed by similar articles [3, 30, 31]. However, similar to El Beshlawy study, [2] we could not confirm this finding.

In our study, alloimmunization was higher in females than males, in agreement with other studies [18, 32–34]. Female gender is an important independent risk factor for alloimmunization and could have a serious impact on female patients in their future life, with pregnancy and delivery exposure adding to their alloimmunization risk. In addition, these patients may have a higher incidence of hemolytic disease of the newborn in their neonates due to their previous alloimmunization.

Alloantibody production in splenectomised patients has been previously reported to be higher than in nonsplenectomised patients. Singer et al. [3] proposed that this could be due to alterations in the RBC membrane which increase immunomodulation causing an enhanced risk of RBC allosensitization. Apart from that, the absence of the spleen’s involvement in eliminating misshaped RBCs could have another crucial role [9]. In our study, there was no statistical significance between splenectomy and the development of alloantibodies similar to what was previously reported [2, 30, 35].

Our research, as well as those of others, has revealed the necessity for measures to prevent the formation of alloantibodies in order to optimise the benefits of transfusion while avoiding complications. In order to reduce the occurrence of RBC alloantibodies and haemolytic transfusion reactions in hemoglobinopathies patients, phenotyping of a newly diagnosed patient, specifically Kell and Rh blood type antigens and transfusion of matched blood components, is crucial. The management of these patients might improve with routine testing for the emergence of alloantibodies in individuals who have received several transfusions and the provision of leukoreduced, Rh and kell phenotyped, antigen-matched blood.

## References

[1] El-Beshlawy A, Youssry I (2009). Prevention of hemoglobinopathies in Egypt. Hemoglobin.

[2] El-Beshlawy A, Salama AA, El-Masry MR (2020). A study of red blood cell alloimmunization and autoimmunization among 200 multitransfused egyptian β thalassemia patients. Sci Rep.

[3] Singer ST, Wu V, Mignacca R (2000). Alloimmunization and erythrocyte autoimmunization in transfusion-dependent thalassemia patients of predominantly asian descent. Blood J Am Soc Hematol.

[4] Asim Qidwai MD, Phil ASM, Phil HMM, Malik F (2018). Trends of red cell alloimmunization in β thalassemia major patients: a single center retrospective study in Karachi. J Blood Disord Treat.

[5] Vaziri M, JavadzadehShahshahani H, Moghaddam M, Taghvaee N (2015). Prevalence and specificities of red cell alloantibodies in transfusion-dependent beta thalassemia patients in Yazd. Iran J Pediatr Hematol Oncol.

[6] Haslina MNN, Ariffin N, Hayati II, Rosline H (2006). Red cell immunization in multiply transfused malay thalassemic patients. Southeast Asian J Trop Med Public Health.

[7] Dimitrios F, John P, Ali T, Maria DC, Michael A, Eleftheriou A (2022). 2021 Thalassaemia international federation guidelines for the management of transfusion-dependent thalassemia. HemaSphere.

[8] Hillyer CD, Blumberg N, Glynn SA (2008). Transfusion recipient epidemiology and outcomes research: possibilities for the future. Transfusion.

[9] Chou ST, Liem RI, Thompson AA (2012). Challenges of alloimmunization in patients with haemoglobinopathies. Br J Haematol.

[10] Ahmed AM, Hasan NS, Ragab SH (2010). Red cell alloimmunization and autoantibodies in egyptian transfusion-dependent thalassaemia patients. Arch Med Sci.

[11] Saifeldeen ER, Awad MA, El-Tonbary YA (2017). Risk for red cell immunization among thalassemic patients. Egypt J Haematol.

[12] Osman NF, Ragab SA, Soliman MA (2017). Alloimmunization in Egyptian children with transfusion-dependent B-thalassaemia: a major challenge. Egypt J Haematol.

[13] Manal A, EID, Marwa MS, Al-Hadad, Ibraheem MB, Maaly MM (2018). Evaluation of Red Cell Alloimmunization in Thalassemic patients with repeated blood transfusion. Med J Cairo Univ.

[14] Politis C, Hassapopoulou E, Halkia P (2016). Managing the patient with haemoglobinopathy and multiple red cell antibodies. ISBT Sci Ser.

[15] Wang L, Liang D, Liu H (2006). Alloimmunization among patients with transfusion-dependent thalassemia in Taiwan. Transfus Med.

[16] Pazgal I, Yahalom V, Shalev B (2020). Alloimmunization and autoimmunization in adult transfusion-dependent thalassemia patients: a report from a comprehensive center in Israel. Ann Hematol.

[17] El Danasoury AS, Eissa DG, Abdo RM, Elalfy MS (2012). Red blood cell alloimmunization in transfusion-dependent egyptian patients with thalassemia in a limited donor exposure program. Transfusion.

[18] Alkindi S, AlMahrooqi S, AlHinai S (2017). Alloimmunization in patients with sickle cell disease and thalassemia: experience of a single centre in Oman. Mediterranean J Hematol Infect Dis.

[19] Murao M, Viana MB (2005). Risk factors for alloimmunization by patients with sickle cell disease. Brazilian J Med Biol Res.

[20] Abu Taha A, Yaseen A, Suleiman S (2019). Study of frequency and characteristics of red blood cell alloimmunization in thalassemic patients: multicenter study from Palestine. Adv Hematol.

[21] Spanos TH, Karageorga M, Ladis V (1990). Red cell alloantibodies in patients with thalassemia. Vox Sang.

[22] Abdelrazik AM, Elshafie SM, El Said MN (2016). Study of red blood cell alloimmunization risk factors in multiply transfused thalassemia patients: role in improving thalassemia transfusion practice in Fayoum, Egypt. Transfusion.

[23] AlDawood RA (2022). The prevalence of cumulative alloimmunization in patients with sickle cell disease at King Fahad university hospital. J Appl Hematol.

[24] Sins JWR, Biemond BJ, van den Bersselaar SM (2016). Early occurrence of red blood cell alloimmunization in patients with sickle cell disease. Am J Hematol.

[25] Allali S, Peyrard T, Amiranoff D (2017). Prevalence and risk factors for red blood cell alloimmunization in 175 children with sickle cell disease in a french university hospital reference centre. Br J Haematol.

[26] Evers D, Middelburg RA, de Haas M (2016). Red-blood-cell alloimmunisation in relation to antigens’ exposure and their immunogenicity: a cohort study. Lancet Haematol.

[27] Chou ST, Alsawas M, Fasano RM (2020). American society of hematology 2020 guidelines for sickle cell disease: transfusion support. Blood Adv.

[28] Pimpaldara RP, Patel AC, Patel J (2015). A study of irregular antibodies in 200 multi-transfused patients. J Evol Med Dent Sci.

[29] Karimi M, Nikrooz P, Kashef S (2007). RBC alloimmunization in blood transfusion-dependent β‐thalassemia patients in southern Iran. Int J Lab Hematol.

[30] Al-Riyami AZ, Al‐Muqbali A, Al‐Sudiri S (2018). Risks of red blood cell alloimmunization in transfusion‐dependent β‐thalassemia in Oman: a 25‐year experience of a university tertiary care reference center and a literature review. Transfusion.

[31] Samarah F, Srour MA, Yaseen D, Dumaidi K (2018). Frequency of red blood cell alloimmunization in patients with sickle cell disease in Palestine. Adv Hematol.

[32] Bashawri LAM (2007). Red cell alloimmunization in sickle-cell anaemia patients. EMHJ-East Mediterr Health J.

[33] Ameen R, Al Shemmari S, Al-Bashir A (2009). Red blood cell alloimmunization among sickle cell Kuwaiti Arab patients who received red blood cell transfusion. Transfusion.

[34] Smith-Whitley K, Thompson AA (2012). Indications and complications of transfusions in sickle cell disease. Pediatr Blood Cancer.

[35] Gupta R, Singh DK, Singh B, Rusia U (2011). Alloimmunization to red cells in thalassemics: emerging problem and future strategies. Transfus Apher Sci.

